# Acceptability of “High sn-2” Infant Formula in Non-Breast Fed Healthy Term Infants Regarding Gastrointestinal Tolerability by Both Parents and Pediatrician: An Open-Label Pilot Study in the Gulf Cooperation Council (GCC) Countries

**DOI:** 10.3390/pediatric13040076

**Published:** 2021-12-01

**Authors:** Mike Possner, Ibrahim El-Neklaway, Mohamed Khater, Mohamed Fikry, Abdeldaem Nazem Alshahoud, Mohamed Salah, Waleed Said, Eslam Tawfik

**Affiliations:** 1 Nestle Nutrition Institute, 60528 Frankfurt am Main, Germany; mike.possner@de.nestle.com; 2 Head of Pediatric Department, Almana General Hospital, Khobar 31952, Saudi Arabia; neklaway@gmail.com; 3 Department of Pediatrics, Faculty of Medicine, Alexandria University, Alexandria 21548, Egypt; drmmkhater@yahoo.com; 4 Department of Pediatrics, Mouwasat Medical Services, Riyadh 13241, Saudi Arabia; 5 Wyeth Nutrition Middle East, Dubai P.O. Box 17327, United Arab Emirates; Mohamed.Ahmed@wyethnutrition.com; 6 Pediatric Consultant, Dr Mohammed Fagih Hospital, Riyadh 13223, Saudi Arabia; drdaemn1968@hotmail.com; 7 Egyptian Academy of Pediatrics, Cairo 11311, Egypt; dr.mohamed.salah@egypediaacademy.com; 8 Head of Pediatrics Department, Al Garhoud Private Hospital, Dubai P.O. Box 36868, United Arab Emirates; drwaleedsaied@hotmail.com; 9 Department of Pediatrics, Faculty of Medicine, Cairo University hospitals, Cairo 12613, Egypt; 10 Sheikh Khalifa Medical City, Abu Dhabi P.O. Box 51900, United Arab Emirates

**Keywords:** breastfeeding, high-sn-2 formula, tolerability, GIT tolerance

## Abstract

Recent studies have highlighted the positive impact of high sn-2 formulas on gastrointestinal (GI) tolerance. We assessed the GI tolerance, acceptability, and safety of high sn-2 infant formula among non-breastfed healthy term infants in the Gulf countries. A multicenter observational study was conducted on 227 healthy-term infants who were prescribed high sn-2 palmitate infant formula and received a minimum of two formula feedings per day for the past two weeks prior to the study’s initiation. The number of stools per day decreased significantly from a median of four (interquartile range [IQR] = 4) at baseline to 3.0 (2) stools per day at the end of follow-up (*p* = 0.015). The percentage of stool amount changed significantly, where 61.2% and 33.7% of the infants had an amount of 25–50% of the diaper and >50% of the diaper, respectively (*p <* 0.001) at the end of the follow-up. Similarly, the percentage of hard stool decreased significantly from 17.4% at baseline to 0.4% of the population at week 12 (*p <* 0.00). The prevalence of colic and abdominal distention declined from 21.4% and 39.9% at baseline to 2.9% and 9.4% at week 12, respectively (*p <* 0.05). The same decline was observed in abdominal distension and regurgitation score (*p* < 0.05).

## 1. Introduction

The optimal food for healthy infants is breast milk, which provides an adequate supply of all essential nutrients for growth and bacterial community to develop infant gut microbiome [1,2]. Around 50% of the dietary calories received from breast milk and most infant formulas are fats, especially triglycerides, which constitute nearly 95% of the milk fats [3]. Palmitate represents about 17–25% of the breast milk fatty acids, and around 70% of it is esterified to the sn-2 (ß) position; however, in infant formula, it is predominantly at the sn-1 and sn-3 positions [3,4,5]. Therefore, the absorption of palmitic acid from human milk is much better than in infant formulas, which can be attributed to the high tendency of palmitic acid in infant formulas to create complexes with dietary minerals and form saponified fatty acids. In return, these complexes can lead to a decrease in both minerals and palmitic acid [6,7,8]. The formation of these complexes is usually associated with poor digestion and harder stools [9]. On the other hand, many reports have mentioned that using formulas similar to human milk’s positional fatty acid profile was associated with better mineral balance, lower fecal calcium excretion, and improved absorption, especially in term and premature infants [6,10].

Therefore, a growing body of evidence has evaluated infant formulas with increased levels of palmitic acid in the sn-2 position to improve infant fat absorption [11]. The high sn-2 palmitate vegetable oil (Betapol^TM^) formula differs from the fat in most infant formulas in that the palmitic acid is predominantly esterified to the sn-2 position of the fat molecule and more closely resembles the palmitic acid in human milk. Fatty acids in the sn-2 position do not readily bind calcium and are easily absorbed [12,13]. Studies have shown that infants who consume a formula containing a mixture of high sn-2 formula and the usual vegetable oils have reduced fecal excretion of calcium fatty acid soaps and softer, more frequent stools [6,10,14]. Moreover, many studies have highlighted the significant difference between the human milk and infant formulas in terms of gastrointestinal tolerance; however, they also showed that this difference is substantially reduced when high sn-2 formulas are used [15,16,17].

According to previous reports, the rate of exclusive breastfeeding in the Gulf Cooperation Council (GCC) countries is low, and a considerable proportion of healthy infants are formula-fed [18,19]. The large variety in the commercially available formulas was noted to confuse parents from the GCC and resulted in frequent changes in the utilized formula [20]. Thus, real-world evidence is critically needed to characterize the outcomes of various infant formulas. Despite high sn-2 formulas exhibiting acceptable efficacy in previous trials, to the best of our knowledge, there are limited data regarding the safety and efficacy of high sn-2 formula in formula-fed and partially breast-fed healthy term infants in GCC countries. Hence, the current prospective study aimed to assess the gastrointestinal tolerance, acceptability by both parents and pediatricians, and safety of high sn-2 infant formula among non-breastfed healthy term infants in the GCC countries.

## 2. Materials and Methods

We prepared the following manuscript in concordance with STROBE guidelines [21].

### 2.1. Study Design and Population

The present study was a multicenter, open-label, observational study that was carried out on healthy-term infants aged between two and 12 weeks through the period from December 2017 to January 2020 in the five centers from the Kingdom of Saudi Arabia (KSA) and the United Arab Emirates (UAE). All infants were recruited through a network of general pediatricians who decided to participate in the study. Healthy-term infants were deemed eligible if they met the following criteria: (1) infants aged two to 12 weeks with normal birth weight (2500–4000 g); (2) infants who were prescribed high sn-2 palmitate infant (Betapol^TM^) formula by their general pediatricians in routine practice as per the recommended feeding table for the age group; and (3) infants who drink at least two formula feedings per day, each to be at least 30 mL. The Betapol^TM^ formula is commercially available for purchase in the two participating countries. Parents or legal guardians of eligible infants were required to sign the written informed consent before initiating any study-related activities. We excluded infants with contraindication to the standard infant formula, an allergy to cow’s milk protein, serious medical or surgical gastrointestinal disease, multiple congenital anomalies, a suspected chromosomal or metabolic disorder, and/or infants with mothers with a health condition or socioeconomic problems that may interfere with their ability to take care of their infants.

### 2.2. Sample Size Calculation

The primary outcome of the present single-arm study was based on estimating the changes in the tolerance parameters at the end of the 12th week of follow-up. For numeric primary outcomes such as volume per stool and stools per week, with 200 infants, the study was able to estimate the mean of such outcomes within a margin of error of about 0.15 of a standard deviation (SD) using 95% confidence intervals. For categorical primary outcomes such as excessive crying, the sample size of 200 infants was able to estimate the prevalence of such outcomes within a margin of error of at most 7% using 95% confidence intervals. Moreover, the study has a power of about 94% to detect changes of about 0.25 SD (as per the study of Kennedy et al., where a change in volume of stool was 0.8 mL, and the SD was 3.1 mL [14]) in the primary numeric outcomes using the paired *t*-test with a significance level of 5%. Assuming that up to 20% dropout rate during the study would be acceptable [22], the study needed to recruit 250 infants.

### 2.3. Data Collection

At the baseline visit, we collected the following data from the parents or legal guardians of eligible infants: age, gender, weight, nationality, concomitant medications, ongoing medications, the use of laxatives, stool characteristic assessed by the Amsterdam stool form scale (including the number of stool per day, percentage of stool amount per the diaper diameter, consistency, color) [23], presence of colic, presence of abdominal distension, regurgitation score, and parents—and physicians—reported tolerability. The percentage of stool amount was assessed by inspection as follows: smear, up to 25% of the diaper, 25–50% of the diaper, and >50% of the diaper. All enrolled infants were followed up for 12 weeks after enrollment to evaluate the change in the stool characteristics, gastrointestinal symptoms, growth parameters, and tolerability by the parents and physicians. The parents received formal training on how to fill the Amsterdam stool form scale and a diary to record the details. A visual explanation of the Amsterdam stool form scale was supplied to the participants to inform them while filling the dairy. Parents and pediatricians were asked to rate the product based on a tolerance assessment scale. The tolerability assessment was divided into poor, acceptable, good, and excellent. All adverse events throughout the study period were recorded. An adverse event was defined as “any untoward medical occurrence in an infant receiving the administered high sn-2 palmitate infant formula, which does not necessarily have a causal relationship with this formula”. A serious adverse event was defined as an event leading to death, life-threatening experience, hospitalization, or persistent disability. All data were recorded in standardized forms, and study investigators received formal training in the data collection process before the study’s imitation.

### 2.4. Study’s Outcomes

The primary endpoints of the present study were the change in the stool characteristics and gastrointestinal tolerance symptoms over the 12-week follow-up period. The secondary endpoints of the present study were the tolerability by the parents and physicians, the changes in the growth parameters over the study’s period, and the incidence of adverse events.

### 2.5. Statistical Analysis

The statistical software SPSSS (IBM SPSS Statistics for Windows, Version 24.0. Armonk, NY, USA: IBM Corp.) was used for data processing and analysis. According to the normality of data distribution, the central tendency and variability of the numerical data were presented in the form of mean ± SD or median with interquartile range (IQR). Categorical variables were summarized by frequency counts and percentages. The hypothesis of significant changes in the number of stools per day and growth parameters over the study’s period was assessed using Wilcoxon’s signed-rank test while the hypothesis of significant changes in the other gastrointestinal tolerance parameters was assessed by the extended McNemar’s test. *p*-value < 0.05 was regarded as statistically significant.

## 3. Results

A total of 276 infants were initially recruited for the present study. Of them, 227 infants (82.2%) completed the study duration. The most common reason for dropout was the loss of follow up (*n* = 42), followed by travel/change in residence (*n* = 4), constipation (*n* = 1), and cow’s milk protein allergy (*n* = 1). Only two infants discontinued the formula, and the rest of the infants maintained the same formula after withdrawal.

### 3.1. Baseline Characteristics

The median age of the infants was 1.22 (IQR = 1.27) months, and 56.5% of them were males. The median weight at enrollment was 4.3 (IQR = 1.13) kg. The Saudi infants accounted for 57.2% of the study cohort, followed by Egyptian (20.7%) and Emirati (6.5%) infants. Fifteen infants (5.4%) were on concomitant medications, mainly in the form of vitamin supplements or medications for relief of colic and constipation. However, there was no use of any laxatives. Only two infants (0.7%) were on a glycerin suppository. Out of the 15 infants, ten had their medication ongoing during the study period (Table 1).

### 3.2. Efficacy Analysis

The changes in the stool characteristics and gastrointestinal symptoms are shown in Table 2. The number of stools per day decreased significantly from a median of 4 (IQR = 4) at baseline to 3.0 (IQR = 2) stools per day at the end of the 12th week of follow-up (*p* = 0.015). However, the change in the number of stools per day from baseline to the end of the 4th week of follow-up was not statistically significant (*p* = 0.679). At enrollment, the percentage of stool amount was 25–50% and 25% in the majority of the infants (56.5% and 16.3%, respectively). These percentages changed with a shift toward higher amounts at the end of follow-up, while 61.2% and 33.7% of the infants had a stool amount of 25–50% and >50%, respectively (*p <* 0.001). Similarly, the percentage of hard stool decreased significantly from 17.4% at baseline to 4.7% and 0.4% of the population at week 4 and week 12, respectively (*p <* 0.001). At baseline, the stool color was mainly a mustard yellow (59.1%). At week 4, the mustard yellow decreased to 50.4%, with an increase in hummus brown (21.4%) and muddy brown (18.1%). At week 12, stool color was mainly hummus brown (47.1%), followed by mustard yellow (31.9%) and muddy brown (14.5%). These changes were statistically significant at both time points (*p <* 0.001).

The gastrointestinal tolerance was another endpoint collected for the study. Colic was prevalent at baseline (21.4%) and declined significantly in weeks 4 and 12 to 4.7% and 2.9%, respectively (*p <* 0.05). The same decline was observed in the incidence of abdominal distension (from 39.9% at baseline to 9.4% in week 12, *p <* 0.05). Furthermore, the regurgitation score showed improvement from 54.7% with no regurgitation at baseline to 65.6% at week 4 and 83.7% at week 12. The change was statistically significant at both week 4 and week 12 (*p <* 0.05).

**Figure 1:**
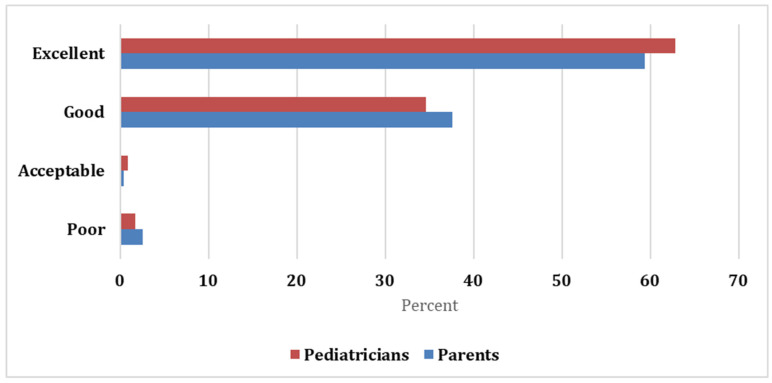
Schemes follow the same formatting.

Both parents and pediatricians gave a favorable assessment, with 59.4% and 37.6% of the parents rating the formula as excellent and good, respectively, while most pediatricians rated the formula as excellent (62.8%) and good (34.6%). Both raters showed excellent agreement with the Kappa statistic = 0.929 and significant *p*-value (<0.05; Figure 1).

### 3.3. Growth Parameters

The median weight of the included infants increased significantly to 5.5 (IQR = 1) and 7 (IQR = 0.9) kg at week 4 and week 12, respectively (*p* < 0.001). The median weight centile also witnessed a significant increase (*p* < 0.05). The height centile decreased from 40.4 (IQR = 53.7) cm at baseline to 37.8 (IQR = 48.4) at week 4, then increased to 39.4 (IQR = 48.6) cm at week 12 (*p <* 0.05). Such a slight change with significant changes might indicate within-subject variability. On the other hand, waist/hip centile and head circumference had the same trend as the weight centile, increasing gradually across the visits, thus reaching statistically significant results (Table 3).

### 3.4. Safety Analysis

Throughout the follow-up period, a total of 29 infants (10.5%) reported adverse events, of which only 20.7% of the adverse events were moderate, and none were severe. The course of these adverse events was uneventful in 25 infants (86.2%), while the remaining four infants had no data regarding the course of the adverse events. Gastrointestinal disorders were the most frequently reported events (*n* = 15); the most common of them was constipation (*n* = 9). Constipation events were reported between the baseline visit and the four week visit. This is consistent with the reported data on stool consistency, which shows that, at 12 weeks, only 0.4% of the children still had hard stools. The second most commonly reported events were infections reported in four infants. Furthermore, two individuals reported conjunctivitis and two upper respiratory infections (Table 4).

## 4. Discussion

The present study sheds light on the safety and efficacy of high sn-2 infant formula in healthy non-breastfed infants. Overall, we demonstrated that the high sn-2 infant formula significantly improved the stool characteristics and the gastrointestinal symptoms by the end of the 12th week amongst healthy non-breastfed infants. Our findings showed a significant reduction in the percentages of hard stool and the infants exhibited a less hard stool at the end of the 12th week of the follow-up. Besides, the percentages of pale yellow, green, and black stools decreased significantly at the end of the study’s follow-up.

These findings are in agreement with those of Yao et al., who found that the infants who received the high sn-2 formula were associated with softer stool, reduced stool soaps, and increased bifidobacteria [24]. Carnielli et al. and Kennedy et al. reported similar findings [10,14]. Kennedy et al. [14] conducted a double-blind, randomized trial (RCT) to estimate the difference between the formula that contained 50% high sn-2 formula and standard formula in term infants. At six and 12 weeks, they showed that the high sn-2 formula was associated with softer stool and reduced proportion of stool soap fatty acids compared to the standard formula. However, they reported no significant difference between both groups in terms of the number of stools per week. In the RCT conducted by Béghin and colleagues [25], they confirmed the efficacy of the high sn-2 formula compared to the control formula in softening the infants’ stool. Another study by Joyce et al. [17] also reported that sn2 palmitate in infant formula decreased stool palmitate soaps and promoted softer stools. It is hypothesized that the main cause behind the softening of stool in infants who received high sn-2 is the reduced saturated fatty acids soaps and replacing it with a structured lipid-containing fat blend enriched in sn-2 palmitate [26,27,28].

Nonetheless, a previous RCT showed no significant difference between the sn-2 formula and the control group in terms of stool consistency [17]. Such findings can be attributed to the low percentage of palmitic acid sn-2 in the formula they used (39% palmitic acid sn-2) compared to 66% used in the study of Carnielli et al. or Kennedy et al. [10,14]. However, this explanation can be challenged by the findings of Litmanovitz et al. [29] in which there were no differences in stool frequency or consistency at six or 12 weeks between the high and low sn-2 palmitate formulas. However, at 12 weeks, fewer infants in the high sn-2 palmitate group had hard stools than the low sn-2 palmitate group (0% vs. 24%) [29]. Thus, further trials are warranted to compare the impact of high and low sn-2 palmitate formulas on the stool characteristics of infants.

Infant colic is a collective term usually used to describe irritability and persistent crying (>three hours per day for >three days per week) by infants. Infants with persistent colic are more prone to improper feeding and poor parental engagement [30]. According to a literature review by Yvan et al., the prevalence of functional constipation in infants was found to be around 15% [31]. The harder stool may contribute to more intestinal pain and infant crying [29]. In the present study, we found that the high sn-2 formula reduced the prevalence of colic and abdominal distention declined from 21.4% and 39.9% at baseline to 2.9% and 9.4% at week 12, respectively. Moreover, more than 83% of the included infants showed no regurgitation after 12 weeks from receiving the high sn-2 formula. In the 2014 study by Litmanovitz et al. [29], it was found that the high-sn-2 formula reduced the percentage of crying infants and the average time spent crying every day. However, these findings should be interpreted cautiously, since it is well-recognized that infant colic improves after three to six months of age [32]; thus, the observed decline in our study may represent a natural course of infant colic, rather than a significant effect of the infant formula.

Regarding the growth parameters, our study showed a significant increase in the weight of the infants as well as the hip/waist ratio and head circumference. The increase in the weight centile in infants who received sn-2 formula can be attributed to the reduced fat-loss compared to the standard formula [24]. According to Carnielli et al. [10], the sn-2 formula was associated with the highest fat absorption compared to other formulas. Interestingly, Zhong and his colleagues [33] showed that the high sn-2 formula had a similar effect to breastfeeding on body growth and prevention of hard stools. Béghin et al. [25], reported that the sn-2 formula and standard formula had a comparable effect on the body mass; however, the sn-2 group was associated with increased bone mineral content. This was further supported by Litmanovitz et al. [34], who showed a significant increase in the bone strength amongst infants receiving a high sn-2 formula that was comparable to human breastfeeding. Consistently, a review by Bar-Yoseph et al. [35] reported that recent studies have confirmed that sn-2 palmitate increases early bone development and mineralization, influences the formation of the intestinal microflora, could lower the severity and extent of intestinal inflammation, and may have neurobiological effects including early infant crying.

In the present study, we showed that the high sn-2 formula was well-tolerable with a low incidence of side effects. A total of 29 infants (10.5%) reported adverse events, of which only 20.7% of the adverse events were moderate, and none were severe. Béghin et al. [25] reported a higher incidence of adverse events (36.8%). However, this percentage was much lower than the control group (46.3%). In the study of Yao et al., they showed that 46.7% of the infants who received sn-2 formula had some adverse events; however, they stated that 93.3% of the adverse events were unrelated to feedings and 83.3% were mild in intensity. The most common reported adverse events were pyrexia, cough, and rhinorrhea [24]. According to the findings of Nowacki et al. [17], there was no significant difference between the sn-2 formula and the control group in terms of the incidence of adverse events (46.4% vs. 42.6%), respectively. Nevertheless, the feeding-related adverse events were only three adverse events.

Such findings were reflected in the acceptability of both parents and pediatricians. In the present study, we found that the high sn-2 formula’s tolerability was rated as good-to-excellent by the pediatricians and parents. In addition to the good safety profile of the sn-2 formula, this high tolerability suggests that the high sn-2 is a good alternative to the standard formulas. Our study has some limitations. First, the risk of selection bias is high in our study. We selected the patients using a convenience sample, and we could not include a control group to estimate the exact efficacy within and between groups. The lack of a control group represents a significant limitation in the present study, as the observed changes in the stool characteristics and other study’s outcomes may be attributed to the natural progress of the infants themselves, rather than the effect of the formula itself. Second, we did not evaluate the stool fat soaps and the bone mineral mass, and third, there was a lack of a breastfed reference group.

## 5. Conclusions

In conclusion, the present real-world data highlight that the high sn-2 infant formula may have a beneficial role in improving the gastrointestinal tolerance and stool characteristics of formula-fed infants. High sn-2 is also tolerable and safe in non-breastfed healthy infants. However, our results should be confirmed by controlled trials with a larger sample size.

## Figures and Tables

**Table 1:** Baseline characteristics of the included infants.

Characteristic	Infants (*n* = 276)
**Age (months)**	
Median (IQR)	1.22 (1.27)
Range	0.46–2.99
**Weight (kg)**	
Median (IQR)	4.30 (1.13)
Range	2.40–7.20
**Male,** No (%)	156 (56.5)
**Nationality**	
Saudi	158 (57.2)
Egyptian	57 (20.7)
Emirati	18 (6.5)
Indian	11 (4)
Jordanian	9 (3.3)
Pakistani	7 (2.5)
Syrian	5 (1.8)
Other	11 (4.0)
**Concomitant Medication**	15 (5.4)
Vitamin D Drops	8 (53.3)
Simethicone Drops	5 (33.4)
Glycerin Suppository	2 (13.3)
**Medication ongoing**	10 (66.7)
**Laxative Use**	0

**Table 2:** Stool characteristics and gastrointestinal tolerance of the infants at baseline, 4 weeks and 12 weeks after administration of “high Sn-2” infant formula.

Endpoint	MD (IQR) Baseline	MD (IQR) Week 4	MD (IQR) Week 12	*p*-Value 1	*p*-Value 2
**Number of stools per day**	4.0 (4.0)	4.0 (3.0)	3.0 (2.0)	0.679 ^a^	0.015 ^a^
	***N* (%) at Baseline**	***N* (%) at Week 4**	***N* (%) at Week 12**		
**Stool Amount**					
Smear	41 (14.9)	5 (1.8)	1 (0.4)		
Up to 25%	45 (16.3)	34 (12.3)	13 (4.7)		
25–50%	156 (56.5)	194 (70.3)	169 (61.2)	0.00 ^b^	0.00 ^b^
>50%	34 (12.3)	43 (15.6)	93 (33.7)		
**Stool Consistency**					
Watery	62 (22.5)	29 (10.5)	25 (9.1)		
Soft	149 (54.0)	170 (61.6)	140 (50.7)		
Formed	17 (6.2)	64 (23.2)	110 (39.9)	0.00 ^b^	0.00 ^b^
Hard	48 (17.4)	13 (4.7)	1 (0.4)		
**Stool Color**					
Mustard Yellow	163 (59.1)	139 (50.4)	88 (31.9)		
Hummus Brown	43 (15.6)	59 (21.4)	130 (47.1)		
Green	18 (6.5)	8 (2.9)	5 (1.8)		
Muddy Brown	11 (4.0)	50 (18.1)	40 (14.5)		
Black	23 (8.3)	4 (1.4)	4 (1.4)	0.00 ^b^	0.00 ^b^
Pale Yellow	18 (6.5)	16 (5.8)	9 (3.3)		
**Colic (excessive crying >3 h/day)**					
Yes	59 (21.4)	13 (4.7)	8 (2.9)	0.00 ^b^	0.00 ^b^
No	217 (78.6)	263 (95.3)	268 (97.1)		
**Abdominal Distention**					
Yes	110 (39.9)	74 (26.8)	26 (9.4)	0.00 ^b^	0.00 ^b^
No	166 (60.1)	202 (73.2)	250 (90.6)		
**Regurgitation score**					
No regurgitation	151 (54.7)	181 (65.6)	231 (83.7)		
Volume equals one coffee spoon (2.5 mL)	74 (26.8)	86 (31.2)	39 (14.1)		
>coffee spoon and <tablespoon (5 mL)	31 (11.2)	9 (3.3)	5 (1.8)		
>tablespoon and <half of the ingested volume	13 (4.7)	-	1 (0.4)		
>half of the ingested volume	7 (2.5)	-	-	0.00 ^b^	0.00 ^b^

^a^ Based on the Wilcoxon signed-rank test. ^b^ Based on the McNemar test.

**Table 3:** Growth Parameters at visit 1 (baseline), visit 2 (week 4), and visit 3 (week 12).

Variable	Median (IQR) at Baseline	Median (IQR) at Week 4	Median (IQR) at Week 12	*p*-Value ^a^
**Weight**	4.3 (1.2)	5.5 (1.0)	7.0 (0.9)	0.000
**Weight Centile**	40.5 (43.8)	42.6 (38.0)	57.4 (34.3)	0.000
**Height**	54 (4.5)	58.4 (4.0)	63.1 (4.0)	0.000
**Height Centile**	40.4 (53.7)	37.8 (48.4)	39.4 (48.6)	0.001
**W/H Centile**	40.7 (54.3)	51.6 (46.2)	65.1 (46.6)	0.000
**Head Circumference**	37 (2.4)	39.4 (1.5)	41.9 (1.5)	0.000
**Head Circumference** **Centile**	55.8 (44.4)	59.0 (38.6)	67.4 (32.0)	0.000

^a^ Based on the Friedman test.

**Table 4:** Incidence of adverse events throughout the period of study.

Type of Adverse Events		Infants (*n* = 276)
**Congenital, familial, and genetic disorders**	Hydrocele	1 (0.4%)
	Obstruction of nasolacrimal duct	1 (0.4%)
	**Total**	**2 (0.8%)**
**Gastrointestinal symptoms**	Colic	1 (0.4%)
	Constipation	9 (3.3%)
	Distention	4 (1.5%)
	Vomiting	1 (0.4%)
	**Total**	**15 (5.4%)**
**General disorders and administration site conditions**	Crying	1 (0.4%)
	**Total**	**1 (0.4%)**
**Hepatobiliary disorders**	Jaundice	1 (0.4%)
	**Total**	**1 (0.4%)**
**Infections and infestations**	Conjunctivitis	2 (0.8%)
	Upper respiratory infection	2 (0.8%)
	**Total**	**4 (1.5%)**
**Irritability**	**Total**	**1 (0.4%)**
**Respiratory, thoracic, and mediastinal disorders**	Nasal sinus blockage	1 (0.4%)
	Wheezy	2 (0.8%)
	**Total**	**3 (1.1%)**
**Skin and subcutaneous tissue disorders**	Dermatitis diaper	1 (0.4%)
	Facial rash	1 (0.4%)
	**Total**	**2 (0.8%)**

## Data Availability

Not applicable.
